# Comparison of the efficiency of digital pathology with the conventional methodology for the diagnosis of biopsies in an anatomical pathology laboratory in Spain

**DOI:** 10.1016/j.jpi.2025.100439

**Published:** 2025-04-01

**Authors:** J.I. Echeveste, L. Alvarez-Gigli, D. Carcedo, Y. Soto-Serrano, M.D. Lozano

**Affiliations:** a Clínica Universidad de Navarra (CUN), Pamplona, Spain; b Hygeia Consulting, Madrid, Spain

**Keywords:** Digital pathology, Conventional methodology, Turnaround time, Pathologist workload, Pending cases, Diagnostic efficiency, Biopsy

## Abstract

**Background/objective:**

Digital pathology (DP) encompasses the digitization of processes related to the acquisition, storage, transmission, and analysis of pathological data, contrasting with conventional methodology (CM) using optical microscopes. This study evaluates the efficiency of DP versus CM in a Spanish pathology department.

**Methods:**

Observational, retrospective, and non-interventional study comparing biopsy samples from 2021 (cases diagnosed using CM) and 2022 (using DP). Variables analyzed were the pathologist who made the diagnosis, the number of slides, and the case area. Outcome efficiency variables were the turnaround-time (TaT), pending cases (active cases each pathologist accumulates daily), and pathologist workload. A significance level of 5% was established, and an exploratory cost-analysis was also performed.

**Results:**

11,922 cases were analyzed: 5,836 and 6,086 diagnosed with CM and DP methodologies, respectively. Mean TaT for CM-diagnosed cases was 10.58 (standard deviation [SD] 7.10) days, compared to 6.86 (SD 5.10) days for DP-diagnosed cases, reflecting a reduction of 3.72 days (*P* < 0.001). With DP, the average reduction in pending cases over a year was around 25 cases, with peaks of 100 fewer pending cases during high workload months. Additionally, DP decreased the pathologist workload by 29.2% on average, with reductions exceeding 50% during peak months.

**Conclusion:**

Our study is the first in Spain to compare the efficiency and costs of DP and CM. DP demonstrated significant efficiency improvements over CM, reducing TaT and pathologist workload. Despite higher initial costs, DP's operational benefits indicate its potential as a transformative diagnostic tool. Further studies are needed to evaluate its long-term cost-effectiveness.

## Introduction

Traditional pathology practices are undergoing a significant transformation towards a digital workflow, marked by the use of computer screens to examine scanned histology slides. Digital pathology (DP) represents a promising alternative in image analysis, substituting manual measurements with computerized techniques and incorporating technologies like whole slide images (WSIs).^1^ WSI, generated by scanning glass slides, facilitate the creation of high-resolution digital images for easy viewing, sharing, and storage. This technological advancement significantly improves the precision and consistency of information extraction from tissue images, mitigating the impact of human subjectivity.^2^

The integration of DP into medical environments represents a transformative advancement towards more efficient and precise image analysis workflows. One primary advantage of DP lies in its potential to significantly enhance diagnostic accuracy and consistency. The digital format empowers pathologists with greater clarity when scrutinizing tissue samples, thereby reducing the risk of misinterpretation. DP offers improvements over traditional microscopy, which may be hindered by issues like slide degradation and limited field of view. Additionally, pathologists can zoom in, rotate, and explore digital slides in detail.^1^^,^^2^

Beyond its diagnostic benefits, DP carries implications for workflow management and collaborative endeavours. Digital slides can be effortlessly archived, retrieved, and shared across the healthcare ecosystem, reducing the physical storage demands associated with glass slides. This not only streamlines collaboration but also facilitates expert consultations across geographic distances, as demonstrated during the COVID-19 pandemic.^3^

Globally, DP is emerging as an innovative technology promising enhanced laboratory efficiency. Many health laboratories and hospitals have successfully implemented DP, overcoming challenges such as high initial costs, workflow adjustments, and resistance from some pathologists.^4^ Despite its widespread deployment, the complete transition to fully digital systems remains limited in some pathology laboratories. Nevertheless, the possible benefits, including healthcare cost reduction, remote case access, web-based consultation, and improved data security, indicate a promising future for DP adoption.^5^ These benefits extend beyond clinical applications to encompass educational and research domains, as well as streamline workload management through tracking, triaging, and assigning cases to specific pathologists.^6^^,^^7^

Considering the costs associated with implementing DP, it is essential to assess opportunities for maximizing return on investment. Costs includes measurable aspects such as shipping expenses, overheads, and staff efficiency as well as less quantifiable factors such as improved communication between pathologists and optimized recruitment.^8^

A systematic literature review performed by the Spanish Health Technology Assessment (HTA) Agency reported that DP is an effective and safe diagnostic technique in clinical practice, with a concordance rate exceeding 95% compared to conventional methodology (CM) diagnosis.^9^

The aim of this study was to compare the efficiency, in terms of turnaround-time (TaT) and cases awaiting diagnosis, of the first year of using DP versus CM in the workflow of the Pathology Department at the Clínica Universidad de Navarra (CUN) in Spain. Additionally, an exploratory cost analysis was performed.

## Methods

### Study design

Observational, retrospective, and non-interventional study conducted under routine clinical practice conditions at the Pathological Anatomy Service of the CUN. Biopsy samples (currently, DP is only utilized for biopsy samples and not for cytologies or necropsies) were divided into two groups according to the diagnostic technique used: cases diagnosed using CM (samples from 2021) and cases diagnosed using DP (technology implemented in January 2022, covering samples from 2022). Therefore, a comparison was made between the two groups (CM and DP).

**Fig. 1:**
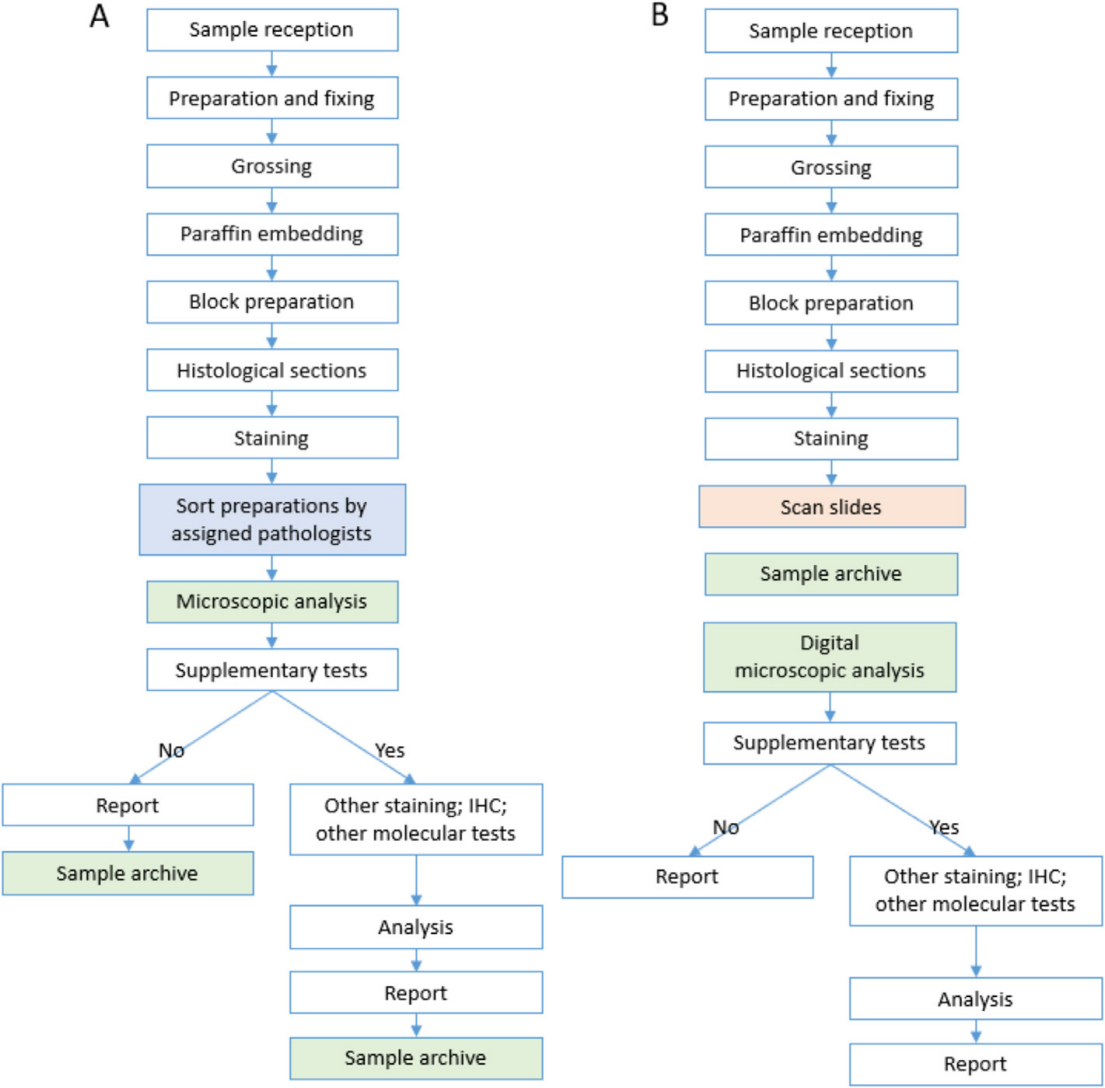
Diagnostic workflow of CM (A) and DP (B) in the Pathological Anatomy Service of the CUN. **Blue:** specific workflow tasks with CM. **Orange:** workflow-specific tasks with PD. **Green:** tasks common to CM and PD but performed at different times of the flow. **White:** tasks common to that do not change between the two flows.

The main difference between CM and DP workflow lies in the post-staining phases: in the CM, technicians and pathologist prepare specimens and perform microscopic analysis directly on the glass slides, while in the DP workflow, the glass samples are digitized by technicians and then archived. Specifically for pathologists, the CM involves the use of optical light microscopes for sample examination and diagnosis (Fig. 1A), whereas DP involves performing digital analysis on a monitor using specialized visualization tools such as Navify (Fig. 1B).

### Study variables

One of the variables used to assess workflow efficiency was the TaT, defined as the time from receipt of the sample to the issuance of the diagnostic report. This outcome variable was assessed according to the pathologist who made the diagnosis (referred to as pathologist A and pathologist B), in relation to the number of slides (1, 2, 3–4, and ≥5 slides) and the case area of the biological sample, categorized as head, neck, and endocrine; dermatopathology; complex digestive; gynaecology and uropathology; neuropathology; and other.

Another efficiency variable was pending cases, defined as the number of active cases (pending diagnosis) each pathologist accumulates daily. For this purpose, a matrix was created for all cases included in the analysis and the 365 days of the year. This enabled the quantification of the daily volume of cases awaiting diagnosis that each pathologist had to deal with throughout the year.

Finally, the pathologist's workload was also analyzed, based on daily pending cases and theoretical daily diagnostic capacity. Thus, the calculated pending cases for each day in 2021 and 2022 were compared with the theoretical number of diagnoses per day (the average number of cases a pathologist should diagnose daily, based on their annual working hours and the total number of cases to be diagnosed in a year). The theoretical workload was calculated considering that approximately 60% of the total working time is spent on case diagnosis.

### Sample size

All biopsy samples from cases assigned to the two pathologists participating in the study (pathologist A and pathologist B) with complete information to meet the main objective of the analysis were included for evaluation. A total of 12,085 clinical cases were initially recorded by the pathologists.

### Statistical analysis

Quantitative variables were described using mean and standard deviation (SD), median, 25th and 75th percentiles, as well as minimum and maximum values. Qualitative variables were analyzed according to absolute and relative frequencies. The TaT of both groups (CM and DP) was compared for the entire sample and stratified by pathologist, area, and number of slides of the case. For the comparisons, *t*-test and Mann–Whitney *U* test were used for continuous variables, while Fisher's exact test and the chi-square test were used for categorical variables, depending on the nature of the comparative variables. A significance of 5% was assumed in all the tests performed. The analysis was made using the R statistical package (version 4.3.1).

### Exploratory cost analysis

The cost of the material resources (laboratory equipment) of the anatomical pathology service of the CUN has been quantified for a period of 5 years from the implementation of the PD in 2021 (analysis period: 2021–2025, both years included), considering a depreciation rate of the equipment of 6 years.^10^^,^^11^ A discount rate of 3% has also been applied to future costs (from 2022 onwards, excluding one-off costs incurred in 2021) (CAPF, 2023).

The equipment used in both workflows (Fig. 1) was not included in the exploratory analysis (i.e., equipment's used in pre-staining tasks or the immunofluorescence microscope). Therefore, only the light microscopes (eight binocular LED microscopes with batteries) are included in the CM workflow and the DP200 scanner (with *uPath* + software, a 10 TB server and algorithms) and workstations (nine computers and monitors) in the PD workflow.

Details of the year of acquisition and the cost of each equipment can be found in Table S1 in the supplementary material.

The exploratory cost analysis was carried out under two alternative scenarios: a scenario reflecting the experience at CUN (replacement scenario), where PD is intended to replace CM (most light microscopes are already amortised and have no cost), and a theoretical scenario (new laboratory scenario), where a pathology department would need to acquire both PD and CM workflow-specific equipment.

## Results

### Samples evaluated

**Fig. 2:**
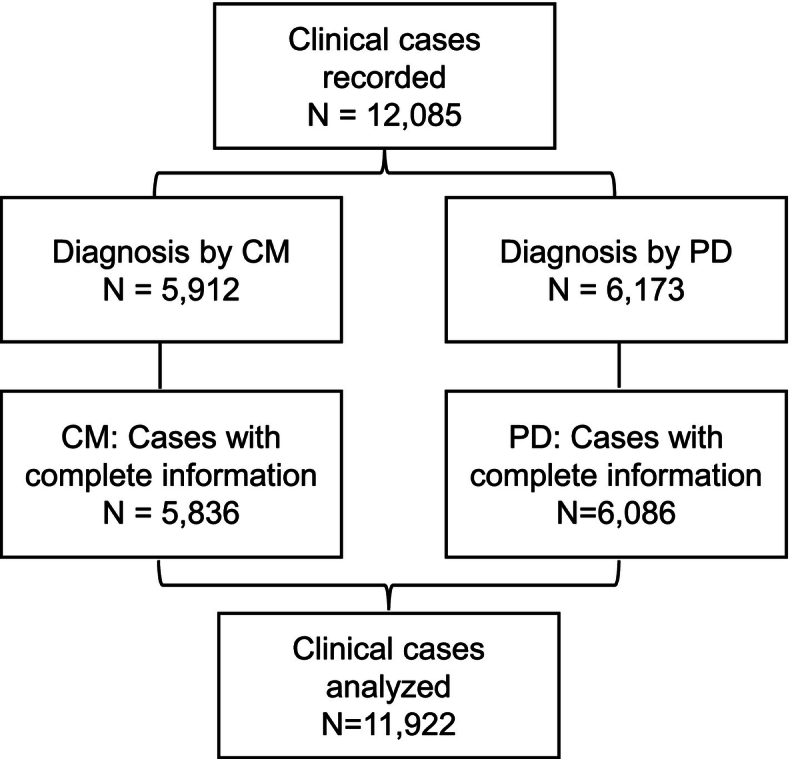
Cases flowchart. **CM:** conventional methodology; **DP:** digital pathology.

A total of 12,085 clinical cases were recorded, of which 5912 were diagnosed using the CM (cases from 2021), and 6173 were diagnosed using DP (cases from 2022). There were 136 cases with missing information on some variable, so 11,922 cases (5836 and 6086 and using CM and DP, respectively) were finally included in the analysis (Fig. 2).

### Descriptive analysis

Figure S1 (Supplementary Material) compares monthly case volumes in our analysis of the CUN pathology department in 2021 (CM) and 2022 (DP). Both years show similar patterns with fewer cases in July and August an increase after the summer.

Regarding the number of slides per case, 36.74% of the cases examined had only 1 slide, 21.56% had two slides, 22.48% had between 3 and 4 slides, and 19.22% had more than 5 slides.

Full descriptions of cases by type (organ of origin) are given in Table S2 in the supplementary material.

### TaT results

The average of the TaT (SD) for all cases included in the analysis was 8.68 (6.44) days. The maximum TaT observed was 55 days for CM and 44 days for PD. The minimum TaT was 1 day, observed in several cases both with CM and PD. A statistically significant reduction in the TaT was observed when using DP compared to CM (mean difference: 3.72 days; median difference: 4.00 days; *P*-value <0.001) (Table 1).

**Table 1:** TaT (days) according to diagnostic techniques (CM and DP).

	CM *N* = 5836	DP *N* = 6086	Difference	*P*-value
Mean (SD)	10.58 (7.10)	6.86 (5.10)	3.72	<0.001
Median (IR)	9.00 [6.00–15.00]	5.00 [3.00–9.00]	4.00	

**CM:** conventional methodology; **DP:** digital pathology; **SD:** standard seviation; IR: interquartile range.

The statistically significant reduction in TaT with PD shown in Table 1 was maintained regardless of the pathologist performing the analysis. A mean reduction of 3.98 days was observed for samples analysed by pathologist A (10.99 versus 7.01 days for CM and PD, respectively) and a mean reduction of 3.45 days was observed for samples analysed by pathologist B (10.16 versus 6.71 days for CM and PD, respectively).

The statistically significant reduction also remains independent of the number of slides per case (Table S3, supplementary material), and independent of case type, although some case areas have small sample sizes (Table S4, supplementary material).

The association between TaT and the number of slides was assessed, and a very low association was found, indicating that, in general, TaT does not seem to depend on the number of slides analysed (Figs. S2 and S3, supplementary material).

### Daily pending cases

**Fig. 3:**
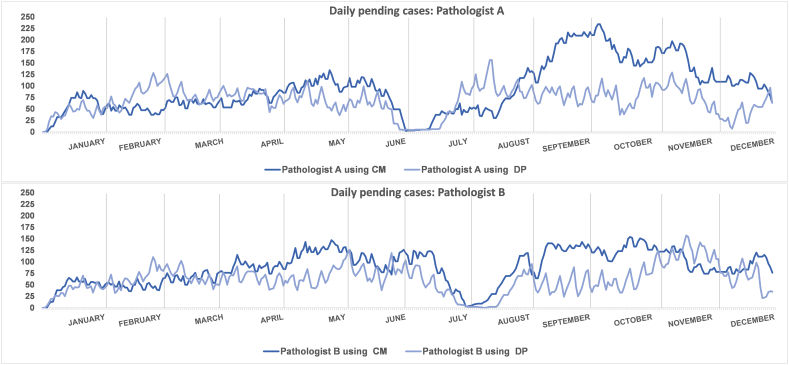
Evolution of daily pending cases for each pathologist (A and B) according to the methodology employed. **CM:** conventional methodology; **DP:** digital pathology.

Fig. 3 shows the daily pending cases over the whole year for pathologists A and B using CM and PD.

Fig. 3 shows that, for both pathologists, the overall backlog of undiagnosed cases is lower with PD (orange) than with CM (blue). In 2021 using the CM, the average number of daily pending cases was 94.48 and 86.99 for pathologists A and B, respectively. In 2022, using PD, the average decreased to 68.48 and 62.33 daily pending cases for pathologists A and B, respectively. However, there are important differences depending on the time of year. No pending diagnostic cases were observed during holiday periods, after which both pathologists experienced workload peaks of over 100 pending cases for several consecutive days.

It was during this post-holiday period that the largest reductions in pending cases were observed for PD compared to CM. For instance, in October 2021 (with CM), peaks of 234 and 154 pending cases were observed by pathologists A and B, respectively. In the same month of 2022 (with PD), the peaks of pending only reached 117 and 99 cases for pathologists A and B, respectively.

### Pathologist workload

**Fig. 4:**
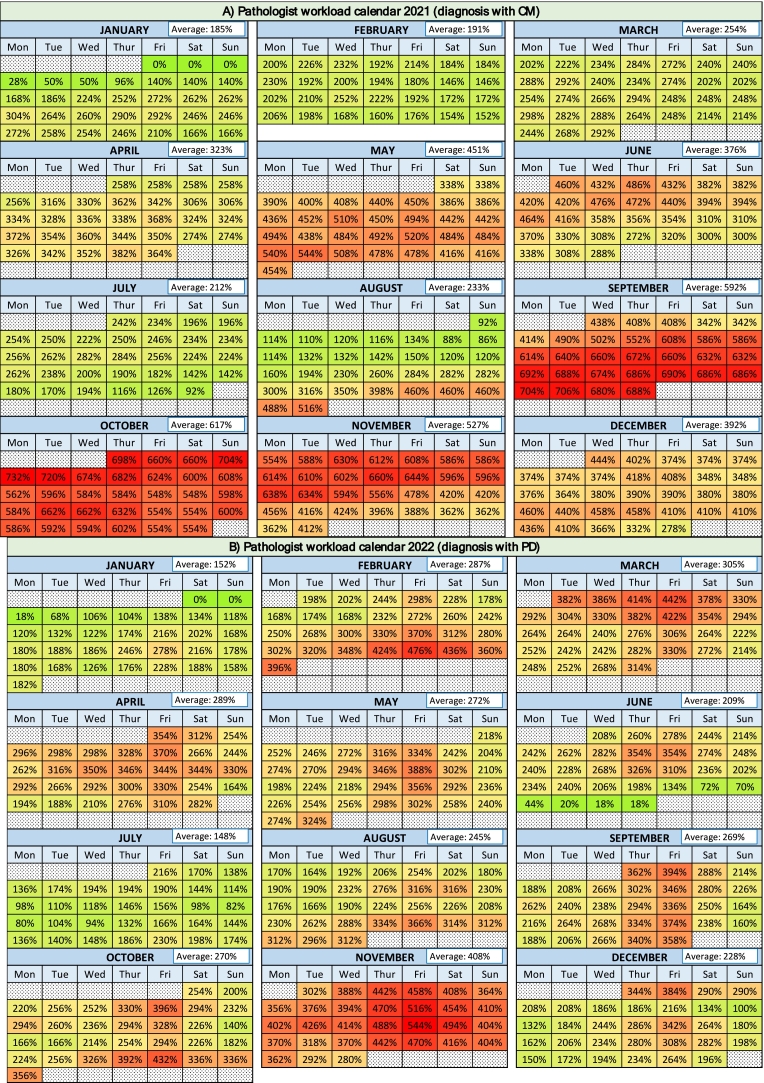
Pathologist workload in 2021 using CM (A) and 2022 using DP (B). **CM:** conventional methodology; **DP:** digital pathology.

A calendar for the years 2021 and 2022 was created to visualize the workload of pathologist A and B (averaged) in terms of their daily pending cases in relation to the theoretical number of diagnoses per day (Fig. 4).

The calendars showed an increase in pathologists' workload in the last semester of both 2021 and 2022, but the introduction of PD seemed to be particularly beneficial during this period. In general (average of the year), PD reduces the pathologist's workload by 29.2% compared to CM, but the workload reduction exceeds 50% in the months of September and October.

### Exploratory cost analysis

In the replacement scenario, the CM workflow equipment costs were €3750 given that only two light microscopes were purchased within the analysis period (2021–2025) and the rest are already amortized. The cost of the PD workflow equipment's was €164,388, resulting in a cost difference of €160,638 over the 2021–2025 (€32,128 annual cost over-run).

In the theoretical new laboratory scenario, CM equipment costs reach €15,000 as all light microscopes would be purchased in 2021, while PD equipment costs remain the same €164,388), resulting in a cost difference of €149,388 over the 2021–2025 period (€29,878 annual cost overrun).

The results of the exploratory analysis should be completed in the future with other types of costs, and compared with the efficiency benefits of PD.

In the present analysis, considering an efficiency variable such as the pathologist workload, it is found that the cost of freeing up 1% of the pathologist workload using DP would cost €1099.

## Discussion

The implementation of DP instead of CM in the diagnostic workflow of the CUN pathology department has demonstrated substantial benefits in both laboratory TaT and operational efficiency in its first year. A significant reduction in TaT is paramount in a clinical setting where timely diagnosis can have a direct impact on patient outcomes. Our results revealed a higher frequency of cases with TaT less than 10 days using DP compared to CM, suggesting that DP not only reduces the average response time, but also brings more cases within a clinically desirable timeframe. This improvement is crucial in managing patient expectations and treatment timelines.

One noteworthy aspect of our findings is the consistency of the reduction in TaT across various several variables, such as pathologist involved, number of slides, and case type. This consistency implies that DP enhances efficiency comprehensively, making it a robust improvement over CM.

The ability to handle diverse case types efficiently is a critical factor for the widespread adoption of DP in different settings. The observed reduction in daily pending cases per pathologist, especially towards the latter part of the year, suggests that DP may alleviate workload pressures, potentially reducing burnout and improving the quality of work for pathologists.

The broader implications of these findings extend beyond mere operational metrics. The reduction in workload intensity and the improved efficiency could enhance pathologist satisfaction and performance, potentially leading to lower turnover rates and higher retention of skilled professionals.^4^ In fact, the two pathologists who participated in the study had subjectively perceived a greater efficiency in the diagnostic process, which was confirmed by the present analysis. Furthermore, they reported no increase in visual fatigue or wrist pain associated with the use of DP, and even noted an improvement in neck discomfort, which could be attributed to a more ergonomic posture compared to conventional microscopes. In any case, in the present study, we only had overall data on the dates of samples entry and report issuance, data that were used to calculate TaT and daily pending cases. Therefore, there are many potential benefits of PD (time saved per task, derived samples, decrease in errors, etc) that could not be included and should be measured in future studies.

Due to this lack of task-specific data, it was not possible to include human resources (laboratory staff) in the exploratory cost analysis and only material resources (equipment) were included. As an exploratory measure, the total cost of human resources for the department was calculated. There were no specific human resources required to implement PD in 2021, so the department's total staffing costs were workflow-independent. For this reason, and in the absence of data on the time saved by each employee using PD, human resource costs were finally not included in the analysis.

Cost-effectiveness analyses of PD to date are scarce, and no studies have been identified that evaluated the efficiency of PD versus CM in comparative studies. Studies conducted by Ho et al. (2014) and Hanna et al. (2019) highlighted the benefits of transitioning to DP, including enhanced productivity, reduced interpretive errors, and cost-savings.^12^^,^^13^ Ho et al. (2021) reported estimated cost-savings of $12.4 million over a 5-year period, attributing the savings to enhanced pathologist productivity and improved workload distribution.^12^ Hanna et al. (2019) documented a 93% decrease in glass slide requests from the department archive, resulting in annual savings of $114,000 in ancillary immunohistochemical orders.^13^ Additionally, they observed an 1-day decrease in TaT for surgical resection cases and an 80% agreement on the improved clinical sign-out experience with WSIs.^13^

In Spain, the literature on PD effectiveness or efficiency is limited to the studies of Temprana-Salvador et al. (2022), Retamero et al. (2022) who describe their experience in implementing PD in their center.^14^^,^^15^ These studies demonstrate successful implementations of DP in healthcare systems, showcasing increased efficiency and collaboration. Temprana-Salvador et al. (2022) described the DigiPatICS project, aimed to deploy DP in an integrative, holistic, and comprehensive way within a network of 8 hospitals in Catalonia. The purpose of the DigiPatICS project was to increase patient safety and quality of care, improving diagnosis and the efficiency of processes in the pathological anatomy departments of the ICS through process improvement, DP, and artificial intelligence.^14^ On the other hand, Retamero et al. (2022) describe the methodology adopted and the resulting experience at Granada University Hospitals in transitioning to full digital diagnosis. Over 160,000 specimens were diagnosed and 800,000 slides were digitised. Notably, they reported scanning errors below 1.5%, affirming the precision of the digitization process.^15^

As highlighted in the systematic review by the Spanish HTA agency, DP offers potential cost-effectiveness and efficiency gains, although long-term data are still needed. The COVID-19 pandemic has accelerated DP adoption, highlighting the need for advanced scanners, training, and integration with existing health systems.^9^

Our analysis is not exempt from certain limitations. The main limitation of this study is its use of a pre-post design over an 1-year period. This approach could be less robust than a parallel-group design, as it does not allow for the observation of long-term evolution or the comparison of the same pathologist's performance over different periods.

In addition, our analysis was limited by the availability of DP data from only two pathologists (hose currently using DP). This limitation meant that we could only analyze the diagnostic areas that these specific pathologists worked on. While our results indicated that the TaT was significantly improved regardless of the diagnostic area, we lacked the TaT data for other areas such as breast or lung cancer.

It should be noted that the analysis included the initial implementation phase of the DP, which includes the learning curve of the laboratory staff. It is likely that the inclusion of the first few months after PD implementation led to an underestimation of the efficiency of PD, particularly in the first half of the year. However, this aspect also provided an opportunity to track the evolution of DP effectiveness over the year, with notable improvements observed towards the end of the study period. This temporal progression highlights the potential for DP to improve diagnostic processes as pathologists become more familiar with the technology.

The calculation of diagnostic workload also presented challenges. In the absence of task-specific data, we estimated pathologists' workload based on the observed number of pending cases every day and a theoretical number of cases that should be daily diagnosed. Although this variable does not accurately capture the pathologist's workload, it gives us an approximation of the percentage of the working day that should be dedicated to diagnosis. This limitation highlights the need for more precise data on active task durations to evaluate potential time savings for pathologists accurately.

Regarding the exploratory cost-analysis, the lack of data on the time spent on each specific task in the diagnostic workflow is a clear limitation. While we have data on the overall benefit to the pathology laboratory of using PD versus CM (TaT, pending cases), it was not possible to quantify the time saved by laboratory staff using PD. For this reason, the cost associated with laboratory staff time was not included in the analysis. This limits the scope of our cost analysis and highlights the need for future analysis to assess how DP could free up professional time. This will allow us to calculate the opportunity cost, which is essential for a comprehensive understanding of the economic impact of DP.

Finally, as mentioned above, it was not possible to conduct a formal cost-effectiveness analysis due to the lack of long-term data or efficacy variables other than TaT or pending cases. Without a long-term perspective, we cannot fully understand the clinical implications of expedited diagnoses facilitated by DP.

In summary, although our study demonstrates significant improvements in TaT associated with the use DP, the limitations described suggest that further research is needed to demonstrate the clinical and economic implications of adopting DP in a pathological anatomy department.

In conclusion, out study, the first performed in Spain comparing DP and CM in terms of efficiency, demonstrate a significant reduction in TaT and a substantial decrease in the pathologist's workload. These results reflect the first year of experience of the anatomical pathology department of CUN after the implementation of PD and the additional costs that this implementation would entail. Future studies are needed to assess the cost-effectiveness of implementing PD and to calculate the year in which the savings in professional time would offset the initial investment.

## Funding

This research did not receive any specific grant from funding agencies in the public, commercial, or not-for-profit sectors. The study has been internally funded by Clínica Universidad de Navarra (CUN).

## Declaration of competing interest

The authors declare that they have no known competing financial interests or personal relationships that could have appeared to influence the work reported in this paper.
