# Supplementary material for: Comparison of the efficiency of digital pathology with the conventional methodology for the diagnosis of biopsies in an anatomical pathology laboratory in Spain
Source: J Pathol Inform. 2025 Apr 1;17:100439. doi: 10.1016/j.jpi.2025.100439 (PMC12088733; doi:10.1016/j.jpi.2025.100439)
Supplement: Supplementary material [file mmc1.docx]

# **Supplementary material**

***Figure S1.*** *Number of cases diagnosed per month in the anatomical pathology laboratory of the CUN in 2021 (using CM) and 2022 (using PD).*

*
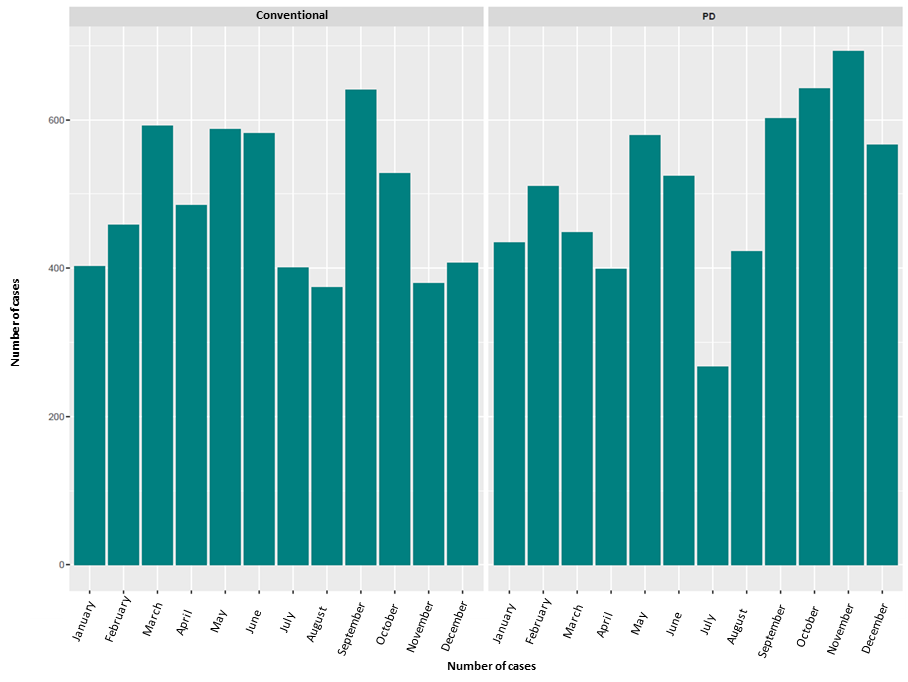
*

***Table S1.*** *Characteristics of the equipment included in the exploratory cost analysis.*

| Materials | Number | Year of acquisition | Unit Cost |
| --- | --- | --- | --- |
| Conventional | | | |
| Battery-powered LED binocular microscope | 8 | 2001 to 2021* | 2,250 € (one-off) |
| DP | | | |
| Scanner DP200 + *uPath*  (+ 2 workstations) | 1 | 2021 | Confidential** |
| Server 10 TB | 1 | 2021 | Confidential** |
| Algorithms | 1 | 2021 | Confidential** |
| Computers | 7 |  | 800.00 € (one-off) |
| Screens | 7 |  | 550.00 € (one-off) |

** one in 2001, one in 2003, two in 2006, one in 2010, one in 2011, two in 2021*

*** 5-year contract*

***Table S2.*** *Number of cases by case area.*

| Case area* | Cases | |
| --- | --- | --- |
|  | n | % |
| Simple digestive (endoscopic biopsies) | 5,179 | 42.32% |
| Dermatopathology | 4,743 | 38.75% |
| Complex digestive (surgical specimen) | 726 | 5.93% |
| Gynecology and Uropathology | 297 | 2.43% |
| Head, neck, and endocrine | 239 | 1.95% |
| Neuropathology | 199 | 1.63% |
| Others** | 856 | 6.99% |
| Total | 12,239 | 100% |

** Hematopathology, bone and soft tissues and lung.

***Table S3.*** *TaT (days) according to the number of slides and the diagnostic technique (CM or DP).*

|  | CM  N=5,836 | DP  N=6,086 | Difference | p-value |
| --- | --- | --- | --- | --- |
| 1 slide | | | | |
| n | 2,059 | 2,321 | - | <0.001 |
| Mean (SD) | 10.47 (6.41) | 6.66 (4.56) | 3.81 (1.85) |  |
| Median (IR) | 9.00 [6.00;14.00] | 5.00 [4.00;9.00] | 4.00 |  |
| Min-Max | [1.00;51.00] | [1.00;44.00] | - |  |
| 2 slides | | | | |
| n | 1,299 | 1,271 | - | <0.001 |
| Mean (SD) | 9.99 (6.53) | 6.21 (4.22) | 3.78 (2.31) |  |
| Median (IR) | 8.00 [5.00;14.00] | 5.00 [3.00;9.00] | 3.00 |  |
| Min-Max | [1.00;43.00] | [1.00;34.00] | - |  |
| 3-4 slides | | | | |
| n | 1,254 | 1,426 | - | <0.001 |
| Mean (SD) | 10.20 (6.72) | 6.63 (4.75) | 3.57 (1.97) |  |
| Median (IR) | 8.00 [6.00;14.00] | 5.00 [3.00;9.00] | 3.00 |  |
| Min-Max | [1.00;41.00] | [1.00;37.00] | - |  |
| ≥ 5 slides | | | | |
| n | 1,224 | 1,068 | - | <0.001 |
| Mean (SD) | 11.76 (8.85) | 8.40 (6.99) | 3.36 (1.86) |  |
| Median (IR) | 10.00 [6.00;16.00] | 6.00 [3.00;11.00] | 4.00 |  |
| Min-Max | [1.00;55.00] | [1.00;44.00] | - |  |

***Figure S2.*** *Scatter plot of the number of slides and the TaT (total cases).*

*
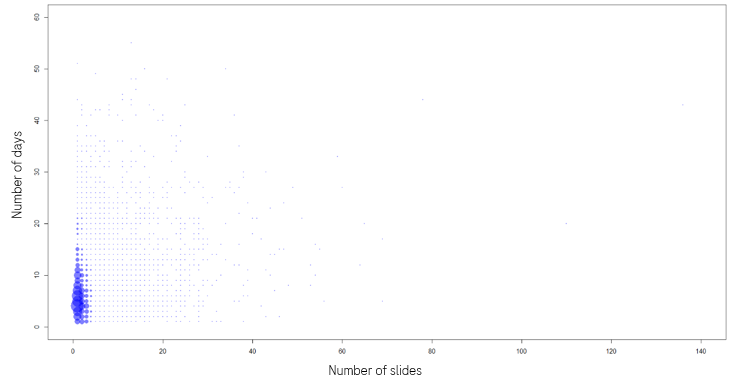
*

***Figure S3.*** *Scatter plot of the number of slides and TaT according to diagnostic technique: CM (A) and DP (B).*


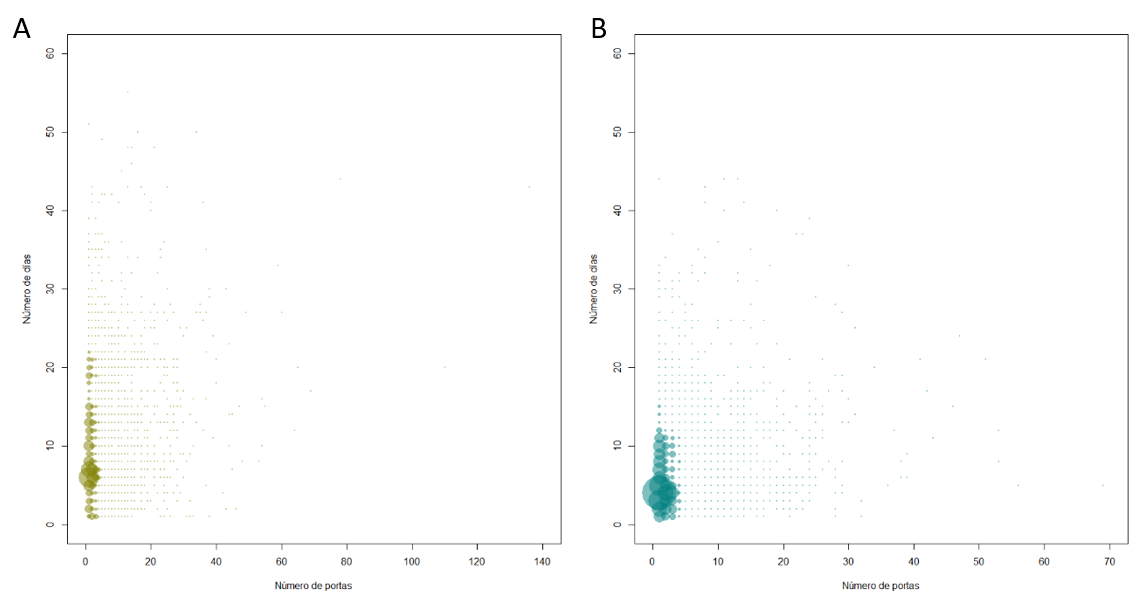


***Table S4.*** *TaT (days) by case area according to diagnostic technique (CM and DP).*

|  | CM  N = 5,836 | DP  N = 6,086 | Difference | p-value |
| --- | --- | --- | --- | --- |
| **Simple digestive (endoscopic biopsies)** | | | | |
| n | 2,445 | 2,734 | - | <0.001 |
| Mean (SD) | 9.83 (6.35) | 5.96 (4.31) | 3.87 (2.04) |  |
| Median (IR) | 8.00 [5.00;14.00] | 5.00 [3.00;9.00] | 3.00 |  |
| Min-Max | [1.00;51.00] | [1.00;43.00] | - |  |
| **Dermatopathology** | | | | |
| n | 2,376 | 2,367 | - | <0.001 |
| Mean (SD) | 10.78 (7.07) | 7.41 (5.40) | 3.37 (1.67) |  |
| Median (IR) | 9.00 [6.00;14.00] | 6.00 [4.00;10.00] | 3.00 |  |
| Min-Max | [1.00;50.00] | [1.00;44.00] | - |  |
| **Complex digestive (surgical specimen)** | | | | |
| n | 406 | 320 | - | <0.001 |
| Mean (SD) | 11.66 (9.63) | 8.16 (6.81) | 3.50 (2,82) |  |
| Median (IR) | 9.00 [5.00;16.00] | 6.00 [4.00;11.00] | 3.00 |  |
| Min-Max | [1.00;55.00] | [1.00;44.00] | - |  |
| **Genecology y Uropathology** | | | | |
| n | 194 | 103 | - | 0.003 |
| Mean (SD) | 12.92 (8.41) | 9.66 (5.50) | 3.26 (2.91) |  |
| Median (IR) | 13.00 [6.00;18.00] | 9.00 [6.00;13.00] | 4.00 |  |
| Min-Max | [1.00;41.00] | [1.00;35.00] | - |  |
| **Head, neck and endocrine** | | | | |
| n | 89 | 150 | - | <0.001 |
| Mean (SD) | 14.62 (7.26) | 9.00 (4.93) | 5.62 (2.33) |  |
| Median (IR) | 15.00 [8.00;21.00] | 9.00 [5.00;12.00] | 6.00 |  |
| Min-Max | [1.00;27.00] | [1.00;28.00] | - |  |
| **Neuropathology** | | | | |
| n | 111 | 88 | - | <0.001 |
| Mean (SD) | 11.83 (6.84) | 8.77 (6.45) | 3.06 (0.39) |  |
| Median (IR) | 10.00 [7.00;15.50] | 7.00 [4.00;11.00] | 3.00 |  |
| Min-Max | [1.00;34.00] | [1.00;33.00] | - |  |
| **Others** | | | | |
| n | 399 | 457 |  | <0.001 |
| Mean (SD) | 12.91 (9.03) | 8.10 (5.94) | 4.81 (3.09) |  |
| Median (IR) | 12.00 [6.00;19.00] | 7.00 [4.00;11.00] | 5.00 |  |
| Min-Max | [1.00;55.00] | [1.00;41.00] | - |  |

***DP:*** *Digital Pathology;* ***SD:*** *Standard Deviation;* ***IR:*** *Interquartile Range.*
